# A bead-based suspension array for the detection of *Salmonella* antibodies in pig sera

**DOI:** 10.1186/s12917-018-1541-x

**Published:** 2018-07-27

**Authors:** Fimme J. van der Wal, René P. Achterberg, Catharina B. M. Maassen

**Affiliations:** 1 Wageningen Bioveterinary Research, P.O. Box 65, 8200 AB Lelystad, The Netherlands; 2 National Institute for Public Health and the Environment (RIVM), P.O. Box 1, 3720 BA Bilthoven, The Netherlands

**Keywords:** *Salmonella*, Serology, Pig, Swine, Bead-based suspension array, LPS, Triazine chemistry

## Abstract

**Background:**

Slaughter pigs are monitored for the presence of the zoonotic pathogen *Salmonella*, using both serology and bacteriology. ELISAs used to investigate pig herds are based on the detection of antibodies against components of the *Salmonella* cell envelope. Nearly all *Salmonella* isolates in food-producing animals are serovars of *Salmonella enterica* subspecies *enterica*, distributed over various serogroups as determined by the composition of their lipopolysaccharide (LPS). ELISAs for *Salmonella* serology are usually based on serogroup B and C1 LPS, often combined with serogroup D or E LPS. Although C2 LPS may improve serology, use of C2 LPS in a broad ELISA was never achieved.

**Results:**

To enable detection of serum antibodies against *Salmonella* in pigs, a bead-based suspension array was developed with five LPS variants (B, 2× C1, C2, D1), each conjugated to a different bead set using triazine chemistry. Reactivity of the beads was confirmed with rabbit agglutination sera and with experimental pig sera. With a mixture of bead sets, 175 sera from slaughter pigs were investigated for the presence of antibodies against *Salmonella*. With a combination of ROC analysis (B and D LPS) and a prevalence estimation based on historic data (C LPS), individual cut-offs were defined for each LPS-conjugated bead set, and assay performance was evaluated.

Results of the suspension array (BC1C1C2D) suggest that more pigs are seroconverted than indicated by a commercial BC1D1-ELISA, and that most of these extra seropositive samples give a signal on one of the beads with C LPS. These results show that expansion of a standard panel with more C LPS variants improves antibody detection.

**Conclusions:**

A suspension array for *Salmonella* serology in pigs was developed, that detects more seropositive sera than ELISA, which is achieved by expanding the panel of *Salmonella* LPS variants, including C2 LPS. The results demonstrate that bead-based suspension arrays allow for testing of pig sera, with the advantage of being able to set cut-offs per antigen. Ultimately, this type of assay can be applied in routine veterinary serology to test for antibodies against multiple *Salmonella* serovars (or other pathogens) in one single serum sample, using up-to-date antigen panels.

**Electronic supplementary material:**

The online version of this article (10.1186/s12917-018-1541-x) contains supplementary material, which is available to authorized users.

## Background

In order to maintain and increase food safety, slaughter pigs are monitored for the presence of zoonotic pathogens such as *Salmonella*. *Salmonella* is a major public health hazard, being responsible for an estimated 28.000 cases of acute gastroenteritis in the Netherlands in 2013, of which around 22% can be attributed to consumption of pork [1]. In Denmark, *Salmonella* control programs started in the early 1990’s and have contributed to a significant reduction of *Salmonella* in pigs and a decrease of salmonellosis [2, 3]. In the Netherlands, a similar program commenced in 2005, based on serology and bacteriology to monitor the *Salmonella* status of pig herds. With the test results, farms are classified according to the percentage of infected pigs, and get advise on prevention and/or measures to reduce *Salmonella* prevalence [4].

The ELISAs used to investigate pig herds for anti-*Salmonella* antibodies are based on the detection of antibodies against components of the *Salmonella* cell envelope. Nearly all *Salmonella* isolates encountered in food-producing animals and products thereof, are serovars of *Salmonella enterica* subspecies *enterica*. Based on the presence of somatic O antigens and flagellar H antigens, a large number of serovars have been described (White-Kauffman-Le Minor scheme) [5]. These are distributed over ~ 30 serogroups as determined by the composition of O antigenic polysaccharides, i.e. the outermost component of LPS. To serotype isolates by their O antigens, *Salmonella* LPS is targeted with specific antibodies in agglutination assays. Conversely, LPS can be used as antigen to probe for anti-*Salmonella* antibodies in animal sera, which is the basis for determining the *Salmonella* status of pig herds using serological ELISAs.

ELISAs based on *Salmonella* LPS from serogroups B and C1 [6, 7] have been used to investigate the infection status of pig herds [8, 9]. In general, serovars belonging to serogroup B, mainly *S*. Typhimurium, are most prevalent in pigs, with a modest contribution of serogroup C and D isolates [1, 2, 10]. Commercial ELISAs are built with at least B and C1 LPS (Prionics, Svanova), and extended variants with D LPS (BioChek, IDEXX) and E LPS (LDL) exist, but C2 LPS is never included, although C2 serovars do occur in pigs and in play a role in human infections (2004–2009 [11], 2010–2016 [12]). It has been suggested that adding C2 LPS may improve serology, but it was shown that in a mixed LPS ELISA this can not be achieved [13].

Over the last few years, several technical solutions have arisen that allow simultaneous testing with multiple antigens, such as bead-based suspension arrays [14]. This type of assay facilitates multiplex serology by appliance of combinations of distinct beads with different antigens to catch specific serum antibodies, that in turn are labelled through fluorescent secondary antibodies. The technology makes use of dedicated equipment to identify the differently coloured bead sets and to detect the acquisition of a fluorescent antibody. It has successfully been used for antibody detection in human serum [15, 16] and also for veterinary applications its feasibility starts being appreciated [17].

The surfaces of the (paramagnetic) beads are modified with carboxyl groups to provide functionality for the chemical coupling to primary amines (e.g. in proteins), for which commonly carbodiimides are used [18, 19]. Cross-linking a nonproteinaceous compound like LPS to carboxylated beads is less common and literature on this subject is sparse. A solution may be provided by triazine cross-linkers that can generate activated esters on carboxyl-groups, which are reactive towards hydroxyl-, amine- and carboxyl-groups [20, 21]. The triazine 4-(4,6-dimethoxy-1,3,5-triazin-2-yl)-4-methylmorpholinium (DMTMM) has successfully been used to cross-link pneumococcal polysaccharides to carboxylated beads [22] and may be the cross-linker of choice to conjugate LPS to carboxylated beads.

The aim of this research was to devise and test a multiplex assay for detecting antibodies in pig serum against *Salmonella* of the most prevalent serogroups. Five LPS variants that represent the *Salmonella* serogroups B, C1, C2, and D1, were conjugated to carboxylated beads with the triazine DMTMM as cross-linking reagent. Using a set of 175 sera from slaughter pigs, the resulting bead-based suspension array was evaluated.

## Methods

### Sera

Various types of sera were used to set-up and evaluate a bead-based serological assay, i.e. agglutination sera from rabbits, experimental pig sera, and sera from Dutch slaughter pigs. Agglutination sera containing the O antigens 4, 5, 6/7, 8, 9, or 12 were obtained from Pro-Labs Diagnostics (BioTrading, Mijdrecht, the Netherlands). Sera from 14 pigs, 42 days after infection or immunization with various *Salmonella* serovars (*S.* Typhimurium, *S*. Choleraesuis, *S*. Infantis, *S*. Goldcoast, *S.* Enteritidis), were kindly donated by M. Swanenburg. Prior to immunization/infection, these sera were all negative for antibodies against *Salmonella* in a commercial BC1D1 LPS ELISA (HerdChek, IDEXX, Hoofddorp, the Netherlands). At day 42 all sera were positive in ELISA, except two *S*. Goldcoast sera (C.B.M. Maassen, unpublished). A set of 175 porcine sera (*n* = 175) was taken from an existing serum set of ca. 1400 sera, of which roughly 15% was seropositive in ELISA (HerdChek, IDEXX, Hoofddorp, the Netherlands; C.B.M. Maassen, unpublished). These sera were collected in December 2003 and the first week of 2004 in a slaughterhouse during slaughter of Dutch pigs. The sera were kept frozen until further use. At the time, the sera were tested for antibodies against *Salmonella* with the commercial BC1D1 LPS ELISA. To ensure sufficient seropositives for evaluation purposes, convenience sampling was conducted by selecting two plates that contained a high number of ELISA positives (*n* = 64; 37%) and therefore the sample set does not reflect the seroprevalence in Dutch pigs. The set contained sera from pigs originating from 10 different farms, with 4 (1 farm) or 15 to 27 (9 farms) sera per farm.

### Preparation of LPS-conjugated beads

To build a suspension array, LPSs of five *Salmonella* serovars of serogroups B, C1, C2, and D1 were used (Table 1), i.e. phenol extracted LPS of *S*. Typhimurium and *S*. Enteritidis (Sigma-Aldrich, Zwijndrecht, the Netherlands), *S*. Livingstone and *S*. Newport (Sussex Research Laboratories, Ottawa, Canada), and TCA extracted LPS of *S*. Choleraesuis, prepared essentially as described [23] and kindly donated by R. van der Hulst-van Arkel. The five LPSs were coupled to five distinct bead sets, using 2.5 × 10^6^ carboxylated paramagnetic beads per bead set in separate coupling reactions (bead addresses 28, 43, 51, 57, or 72; MagPlex Microspheres, Luminex, ‘s-Hertogenbosch, the Netherlands) with the triazine DMTMM (Sigma-Aldrich) as described [22].

**Table 1:** O-antigenic formulas of used LPS

Serovar	O antigens	Serogroup
*S.* Typhimurium	1, 4, 5, 12	B (O:4 group)
*S*. Choleraesuis	6, 7	C1 (O:7 group)
*S.* Livingstone	6, 7	C1 (O:7 group)
*S.* Newport	6, 8	C2 (O:8 group)
*S.* Enteritidis	1, 9, 12	D1 (O:9 group)

List of O antigens of five *Salmonella* serovars of which purified LPS was coupled to beads. Phage dependent O antigens are not listed, except O:1 for *S*. Enteritidis, and the corresponding serogroups are given with the O group designations between brackets [5]

### Bead-based serology

Suspension arrays were performed with a flow cytometry-based Luminex 200, using LPS-conjugated paramagnetic beads to probe for antibodies in agglutination serum (rabbit) or pig serum; sera were diluted respectively 1:25 or 1:200 in phosphate buffered saline with elevated levels of NaCl (1 M). Serology was performed with a suspension array as described [24], i.e. per assay 1000 beads of each bead set were mixed with diluted serum. Serum antibodies bound to LPS-conjugated beads were fluorescently labelled using biotinylated Protein AG [24] (rabbit sera) or biotinylated anti-pig secondary antibodies (1:5000; Jackson ImmunoResearch, Newmarket, United Kingdom) (pig sera) in combination with streptavidin-phycoerythrin (1:2000; SNN1007, Invitrogen, Life Technologies, Bleiswijk, the Netherlands). The fluorescence of at least 100 beads per bead set was measured and the resulting signal was expressed as median fluorescence intensity (MFI).

### Experiments

To determine if LPS-conjugated beads were able to detect O antigen specific antibodies, five different LPS-conjugated bead sets were tested with agglutination sera that contain antibodies against the *Salmonella* antigens O:4, 5, 6/7, 8, 9, or 12. To evaluate the ability of the LPS-conjugated beads to detect anti-*Salmonella* antibodies in porcine sera, the five bead sets were mixed and tested in a multiplex assay with sera from 14 pigs immunized or infected with various *Salmonella* serovars: *S*. Typhimurium (3), *S*. Choleraesuis (3), *S*. Infantis (2), *S*. Goldcoast (3), *S*. Enteritidis (3) (M. Swanenburg, unpublished). A set of 175 sera from Dutch slaughter pigs were tested with the fiveplex suspension array and the results were compared to those obtained by a commercial BC1D1 LPS ELISA.

### Cut-off values

In order to analyse sera from slaughter pigs, cut-offs were defined for each LPS-conjugated bead set. For beads with B or D LPS and for beads with the C LPS variant different approaches were chosen to define cut-offs.

Since most seropositive sera will have antibodies against B LPS [11], and B and D LPS share O antigens (see Table 1), it is likely that most of the 64 (BC1D1 LPS) ELISA positive sera will react on beads with B and/or D LPS. So, cut-offs for the B and D1 LPS bead sets were established by performing ROC analyses (GraphPad Prism 5.04, GraphPad Software, San Diego, California, USA).

Since only a limited number of sera are expected to have antibodies against C1 or C2 LPS, and the BC1D1 ELISA does not return individual results for C1 or C2, ROC analyses are not possible. Therefore, for each C LPS bead set, the average signal of a number of estimated seronegatives plus three times the standard deviation [25] was taken as cut-off value. Estimation of the number of seronegatives for antibodies against C LPS was performed using historic data on 2520 serotyped *Salmonella* pork isolates from 2004 to 2009 [11]. The prevalence of serotypes in the 2004–2009 isolates was projected on the set of 175 sera. Of the isolates, resp. 60.4, 11.1, 7.8 and 4.4% belonged to serogroups B, C1, C2 or D1, and 16.3% belong to other serogroups [11]. Assuming a similar frequency in the 64 ELISA-positive sera (i.e. positive on B, C1 or D1 LPS), it can be expected that of the 64 ELISA positive sera a fraction of C1 / (B + C1 + D) may have antibodies against *Salmonella* from the C1 serogroup, i.e. ca. nine sera (9.4). Antibodies against C2 LPS are only partly detected by the BC1D1 ELISA, so, the fraction of the 175 sera that could carry antibodies against C2 LPS needs to be derived from the previous calculations. As 7.8/11.1 is the ratio of C2 to C1 serovars in the historic data, in the 175 sera seven sera (6.6) could be positive for C2. Based on the estimation that the 64 ELISA positive sera, and in fact the whole set of 175 sera, may contain nine C1 positive sera, it is likely that the larger part of the 175 sera will be seronegative for C1. Based on the estimation that the set of 175 sera set may contain only seven C2 sera, it is likely that the larger part of the 175 sera will be seronegative for C2. For both the C1 and C2 beads, arbitrarily the 150 lowest signals were defined as seronegative.

### Data analysis

Once cut-offs had been defined for each bead set, the cumulative outcomes of (different combinations of) bead sets were determined. Sera were designated seropositive when for at least one of the bead sets a signal was acquired above the corresponding cut-off. These designations were used to evaluate assay performance in simple 2 × 2’s. Only for combinations of bead sets were cutoffs were determined by ROC analysis (B and D LPS), (relative) specificity, (relative) sensitivity, efficiency, and Cohen’s kappa, in comparison with ELISA, were determined as described [26].

## Results

### Detection of anti-*Salmonella* antibodies in agglutination sera

**Fig. 1:**
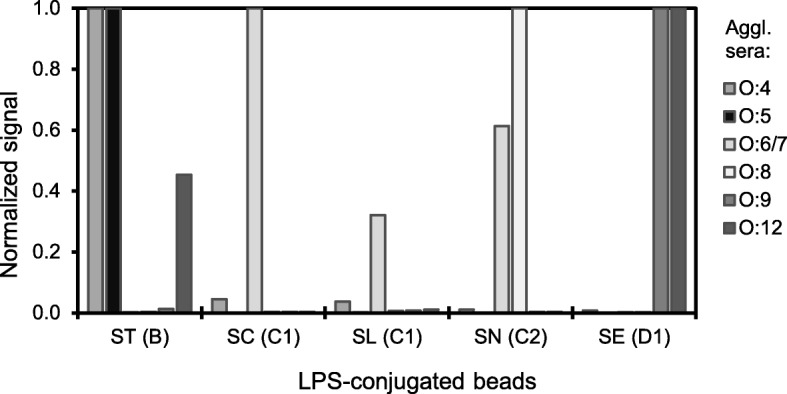
Detection of anti-*Salmonella* antibodies in agglutination sera with the fiveplex suspension array. Five bead sets, each conjugated with *Salmonella* LPS as indicated on the x-axis, were used to detect anti-*Salmonella* antibodies in six agglutination (rabbit) sera, specific resp. for O:4, 5, 6/7, 8, 9, and 12. For each bead set, results are given for six agglutination sera, normalized to the peak value. The five beads were tested in a multiplex assay, but results are presented per bead set. Abbreviations: ST, *S*. Typhimurium; SC, *S*. Choleraesuis; SL, *S*. Livingstone; SN, *S*. Newport; SE, *S*. Enteritidis

The signals obtained on each bead set with the respective agglutination sera (Fig. 1, normalized signals) were in concordance with the LPS present on each bead sets (see list of O antigens in Table 1) and ranged from 405 to 7308 MFI. Other signals ranged from 3 to 17 MFI, i.e. signals of agglutination sera on beads carrying LPS with disparate O antigens, as well as signals of negative control samples on all five bead sets, namely agglutination serum against O:20 or no serum at all (not shown). These results indicate that the antigenic properties of LPS are retained when a triazine is used to immobilize LPS on carboxylated beads, and that the resulting LPS-conjugated beads can be used to detect O antigen specific antibodies.

### Detection of anti-*Salmonella* antibodies in experimental pig sera

**Fig. 2:**
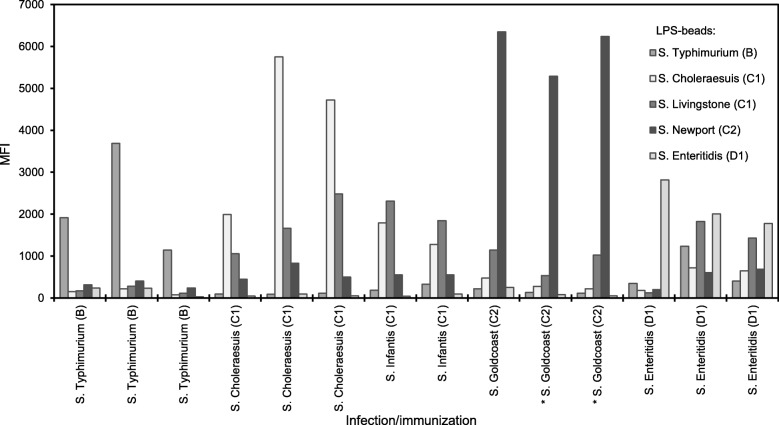
Detection of anti-*Salmonella* antibodies in experimental pig sera with the fiveplex suspension array. The fiveplex suspension array was evaluated using 14 sera of pigs infected or immunized with various *Salmonella* serovars, as indicated on the x-axis. For each serum, results are given for five LPS-conjugated bead sets. All sera were positive in a BC1D1 LPS ELISA, except the two *S*. Goldcoast sera indicated by an asterisk (C.B.M. Maassen, unpublished). Signals are given in MFI (median fluorescence intensity)

The results of the suspension array with experimental pig sera showed that each serum is recognized by LPS of the corresponding serogroup, i.e. B sera are recognized by B LPS, C1 sera by the two C1 LPS variants, C2 sera by C2 LPS, and D1 sera by D1 LPS (Fig. 2). Sera with antibodies against *S.* Choleraesuis (C1) had more affinity for C1 LPS from *S.* Choleraesuis than for C1 LPS from *S.* Livingstone, whereas *S*. Infantis (C1) sera preferred C1 LPS from *S.* Livingstone over C1 LPS from *S.* Choleraesuis. All C1 sera reacted only weakly with C2 LPS, but C2 sera (*S.* Goldcoast) reacted very well with C2 LPS (from *S*. Newport), and much weaker, or not, with the C1 LPS variants. Importantly, two of the C2 sera, that are negative in the BC1D1-ELISA, gave high signals on C2 LPS. Of the three D sera, one cross-reacted with B LPS and two reacted also with C LPS.

Taken together, these results showed that, apart from some cross-reactions of D1 sera, the responses on all LPS-conjugated bead sets matched the expected presence of O antigen specific antibodies in the experimental sera, and that the application of C2 LPS enables additional detection of porcine antibodies against C2 *Salmonella* serovars.

### Detection of anti-*Salmonella* antibodies in sera from slaughter pigs

Results of the suspension array with sera from slaughter pigs were analysed as described in Methods (see Additional file 1: Table S1 for results). Cut-offs for B and D1 LPS-conjugated bead sets were based on ROC analyses using the BC1D1 LPS ELISA as criterion (Table 2). For the bead set with B LPS a cut-off of 1713 MFI was chosen, i.e. specificity was prioritized over sensitivity to keep the number of false-positives (in comparison with ELISA) low. For the bead set with D1 LPS it was not possible to extract a cut-off at which both sensitivity and specificity were higher than 90% (not shown). Since prevalence of D1 serovars in Dutch pigs from the selected period is low, specificity was prioritized over sensitivity and a cut-off of 1105 MFI was chosen. Cut-offs for the three bead sets with C LPS were estimated based on historic prevalence data, designating the 150 lowest signals on each bead as seronegative, and in turn using these to calculate cut-offs [25] (Table 3). Notably, the cut-off for beads carrying C2 LPS was relatively high. For different constellations of LPS-conjugated beads, assay performance was compared with the ELISA (Table 4), showing that the number of positives in bead-based serology increased when LPS variants are added to the test panel. When all five results are taken into account, this result in 78 seropositives by suspension array, out of a set of 175 pig sera in which ELISA detects 64 seropositives.

**Table 2:** Cut-offs of the two bead sets with B and D1 LPS

**Beads with** ***S.*** **Typhimurium LPS (B) compared with a commercial BC1D1 ELISA**							
Relative sensitivity (%)	90.6	92.2	93.8	95.3	96.9	98.4	100
Relative specificity (%)	98.2	97.3	93.7	93.7	91.9	63.7	62.2
Cut-off (MFI)	1713	1668	1453	1406	1311	636	617
E+ Bb- (FN)	6	5	4	3	2	1	0
E- Bb + (FP)	2	3	7	7	9	40	42
Bb+	60	62	67	68	71	103	106
**Beads with** ***S.*** **Enteritidis LPS (D1) compared with a commercial BC1D1 ELISA**							
Relative sensitivity (%)	65.6						
Relative specificity (%)	100						
Cut-off (MFI)	1105						
E+ Db- (FN)	22						
E- Db + (FP)	0						
Db+	42						
E+ Bb- Db+	2						

Data of 175 sera from slaughter pigs were subjected to ROC analysis using the results of a BC1D1 LPS ELISA as criterion (111 ELISA negatives, 64 ELISA positives). For the bead set with B LPS (upper table), specificities and cut-offs are given for sensitivities ranging from 90 to 100%. In addition, the numbers of false negatives (E+ Bb-), false positives (E- Bb+) and the total of positives on the B beads (Bb+) are given. For the bead set with D1 LPS (lower table), for 100% specificity the corresponding sensitivity and cut-off are given, as well as the numbers of false negatives (E+ Db-), false positives (E- Db+), the total of positives on the D beads (Db+), and the number of ELISA positives that are negative on B beads but positive on D1 beads (E+ Bb- Db+). As the used ELISA is *assumed* to be the perfect test, sensitivity and specificity related to this ELISA are referred to as ‘relative sensitivity’ and ‘relative specificity’. Abbreviations: E+, ELISA positive; E-, ELISA negative; Bb+, positive on B beads; Bb-, negative on B beads; D+, positive on D1 beads; D-, negative on D1 beads, FN, false negative; FP, false positive

**Table 3:** Cut-offs of the three bead sets with C LPS

	SC (C1)	SL (C1)	SN (C2)
Average MFI	419	439	1389
SD	215	212	739
Cut-off (MFI)	1064	1074	3607

Based on historic data [11] it was estimated that responses on bead sets with C LPS by C specific antibodies would not be among the 150 lowest signals from the set of 175 sera. So, for each C LPS bead set, the 150 lowest signals (presumed negatives) were used to calculate cut-offs, defined as the sum of averaged MFIs plus three times the standard deviation. Abbreviations: SC (C1), beads with C1 LPS from *S*. Choleraesuis; SL (C1), beads with C1 LPS from *S.* Livingstone; SN (C2), beads with C2 LPS from *S.* Newport

**Table 4:** Performance of combinations of bead sets in comparison with a commercial BC1D1 ELISA

		B beads				
		pos	neg	total	Relative sensitivity	91%
ELISA (BC1D1)	pos	58	6	64	Relative specificity	98%
	neg	2	109	111	Efficiency	95%
	total	60	115	175	Cohen’s kappa	0.90
		B + D1 beads				
		pos	neg	total	Relative sensitivity	94%
ELISA (BC1D1)	pos	60	4	64	Relative specificity	98%
	neg	2	109	111	Efficiency	97%
	total	62	113	175	Cohen’s kappa	0.93
		B + D1 + C1 + C1 beads				
		pos	neg	total		
ELISA (BC1D1)	pos	61	3	64		
	neg	13	98	111		
	total	74	101	175		
		B + D1 + C1 + C1 + C2 beads				
		pos	neg	total		
ELISA (BC1D1)	pos	61	3	64		
	neg	17	94	111		
	total	78	97	175		

For four different combinations of LPS-conjugated bead sets, the results on individual bead sets were used to assign a serostatus to each of the 175 tested sera from slaughter pigs. Sera were designated seropositive when a signal above the threshold was detected with at least one of the bead sets. The resulting designations were used to determine the performance of the various bead set combinations. Only for B and D beads (see Methods) relative sensitivity, relative specificity, efficiency, and Cohen’s kappa were determined [26], using the BC1D1 ELISA as criterion

## Discussion

This study describes the development of an immunoassay for the detection of anti-*Salmonella* antibodies in porcine sera that may be helpful in the existing *Salmonella* control programs [2–4]. *Salmonella* serology focuses on serovars of the B1, C1 and D1 serogroups. Although C2 LPS shares the O:6 antigen with C1 LPS, C1 LPS is not sufficient to detect C2 specific antibodies [7, 13] and attempts to include C2 LPS in ELISA with mixed LPS failed. Nevertheless, inclusion of C2 LPS is relevant as nearly 8% of isolates encountered in Dutch slaughter pigs in 2004–2009 were C2 serovars [11]. The goal of this research was to develop a multiplex assay for the detection of antibodies in pig serum against *Salmonella*, including C2 serovars, that is suitable for serological monitoring of the *Salmonella* status of pig herds.

To develop true multiplex serology with separate read-outs for each LPS variant, the principle of a bead-based suspension array was adopted. Apart from facilitating multiplex serology, such arrays are very flexible as they are amenable to adaptation and expansion, simply by addition of extra bead sets with new antigens. Additional benefits are that antigen immobilization can be performed under optimal conditions for each antigen in the absence of other antigens, that for each antigen/bead combination a separate cut-off can be defined, and that the paramagnetic nature of the beads allows for automation of procedures [14, 17].

The first hurdle was to immobilize LPS on beads. For ELISAs, antigens are often immobilized by passive adsorption [6, 27], but for suspension arrays covalent coupling is required. For this, beads are available that are functionalized with carboxyl groups to conjugate compounds with their primary amines using carbodiimide chemistry. Many protocols and principles [28–32] tested prior to this study did not result in stable beads, in contrast to a published procedure to conjugate polysaccharides using the triazine DMTTMM [22]. This method was chosen to conjugate each *Salmonella* LPS variant to a different carboxylated paramagnetic Luminex bead set with a characteristic fluorescent signature.

The LPS-conjugated bead sets were evaluated with agglutination sera to demonstrate that upon immobilization with DMTMM the antigenic properties of *Salmonella* LPS were retained (*cf*. Fig. 1). With sera from experimentally infected / immunized pigs it was shown that the immune response induced against *S*. Typhimurium, the most prevalent B serovar in pigs, resulted in antibodies primarily against B LPS (O:1, 4, 5, 12), not against D1 LPS (O:1, 9, 12). This may suggest that for pigs the O:4 and 5 antigens are immunodominant over O:1 and/or O:12. Three experimental D1 sera however did react with B LPS, suggesting pigs infected with Salmonella from the D1 serogroup also produce antibodies against the O:1 and/or O:12 antigens that are shared with B LPS. Two experimental D1 sera also reacted with C LPS without the existence of shared specific O antigens; an explanation was not found, but pre-existing antibodies were not found in sera from day 0 of these animals in a BC1D1 ELISA (C.B.M. Maassen, unpublished). Of the experimental C1 sera, signals on homologous LPS was very high (*S.* Choleraesuis) compared with signals on non-homologous C1 LPS, which may suggests that optimal assays should include LPS of circulating serovars. The response of C2 sera however was low on C1 LPS and high on C2 LPS (cf. Fig. 2), as reported in literature [7, 13]. Importantly, two of the C2 sera positive in the suspension array were negative in the BC1D1 LPS ELISA. This observation justifies the inclusion of C2 LPS in the antigen panel and demonstrates the power of multiplex assays that allows interrogation of serum samples with LPS antigens that are not mixed but physically separated, and yet probe for various antibodies in one single serum sample. Using the modest set of 14 experimental sera, there was no sign of erratic responses on C2 LPS, as reported earlier for mixed LPS ELISAs with C2 LPS [13]. However, with sera from slaughter pigs a relatively high background on C2 beads were observed (Additional file 1: Table S1), which was dealt with by assigning individual cut-offs for each LPS conjugated bead set, showing the power of bead-based suspension arrays in case different cut-offs per antigen are required.

For evaluation purposes, a serum set from Dutch slaughter pigs from 2003 to 2004 was chosen that purposely contained a high number of seropositives (37% in ELISA) which does not reflect the seroprevalence in Dutch pigs; in a baseline survey seroprevalence was estimated to range from 6.5 to 7.9% in 2006–2007 [33]. To compare tests, individual cut-offs are required per bead set, i.e. per LPS, for which a two-way approach was used. For B and D sera an ROC analysis was performed, for C beads cut-offs were estimated using historic data on serotyped porcine *Salmonella* isolates. In case of herd monitoring, assay specificity may be prioritised over sensitivity, thereby permitting a certain number of false negatives. So, to limit the number of false positives, but without sacrificing too much sensitivity, for B beads a cut-off was selected that resulted in a relative specificity of 98.2% (two false positives) and a relative sensitivity of 90.6% (six false negatives) in comparison with the BC1D1 ELISA. As most ELISA positive pigs will have an immune response against B, this approach was acceptable to find a cut-off of the B beads, but for D beads this was difficult as only a limited number of pigs will have antibodies against D LPS. So, here it was important to select a cut-off that resulted in 100% relative specificity, with no false positives at all. By using the corresponding cut-off, two sera were found positive on D1 beads (that were negative on B beads) that were also positive in the BC1D1 ELISA, showing the contribution to the suspension array of D1 LPS in the antigen panel.

The cut-offs for beads with C LPS were based on an estimation using a historic data set. For each bead set with C LPS the cut-off was based on the signal of (presumed) 150 negative sera plus three times the standard deviation [25], which is only appropriate if the assumption is true that the prevalence of sera with antibodies against C LPS is low. In fact, with so many sera designated seronegative, the cut-off will be high, which should be beneficial for the assay specificity (not the sensitivity) [25]. By doing so, in this evaluation 17 ELISA negative sera are positive on one of the beads with C LPS, of which five exclusively on C2 LPS. The approach chosen to calculate cut-offs for beads with C LPS will result in relatively high cut-offs, yet several C seropositives were detected in de suspension array that are seronegative in ELISA. These results show that the suspension array detects more C1 positive sera than the ELISA, maybe because of the selected C1 serovars. The results further show that inclusion of C2 LPS also improves *Salmonella* serology, but this is only possible because separate cut-offs can be defined; the cut-off for beads carrying C2 LPS was more than three times higher than cut-offs of the C1-beads. Although a limited data set was used and cut-offs for the resulting signals had to be estimated using historic data, the results of the BC1C1C2D-suspension array suggest that more pigs are seroconverted than indicated by the BC1D1-ELISA. As most of these are positive in the suspension array on one of the C LPS variants, the results clearly show that expansion of a standard BC1D1 panel with more C LPS variants improves antibody detection. Future improvements could include updates with LPS of circulating serovars to create large panels for improved serology.

## Conclusions

This study is a successful first exploration into multiplex detection of antibodies against *Salmonella* in pigs. A bead-based suspension array for *Salmonella* serology in pigs was developed using the triazine DMTMM to covalently link LPS to Luminex beads. The results show that bead-based suspension arrays allow for testing of sera without adverse effects of mixing LPS, and demonstrate the amenability of bead-based suspension arrays for straightforward expansion (or revision) of antigen panels, using individual cut-offs for each antigen. The resulting suspension array is capable of detecting more seropositive pig sera than a commercial ELISA, nonetheless, a validation with a large set of randomly selected field sera would still be required. Ultimately, this type of assay can be applied in routine veterinary serology to synchronously probe for antibodies against multiple pathogens/antigens in one single serum sample.

## Additional file

Additional file 1: **Table S1.** Raw data and interactive cut-offs. MFI data for each LPS beads set for 175 pig sera. Data are protected and can not be changed, the cut-offs however can be manipulated. (XLSX 61 kb)

## Abbreviations

DMTMM: 4-(4,6-dimethoxy-1,3,5-triazin-2-yl)-4-methylmorpholinium
ELISA: Enzyme-linked immunosorbent assay
H antigen: Flagella
LPS: Lipopolysaccharide
MFI: Median fluorescence intensity
O antigen: Lipopolysaccharide
ROC: Receiver operating characteristic
SC: *S*. Choleraesuis
SE: *S*. Enteritidis
SG: *S*. Goldcoast
SI: *S*. Infantis
ST: *S*. Typhimurium
E +: ELISA positive
E-: ELISA negative
Bb +: Positive on B beads
Bb-: Negative on B beads
D +: Positive on D1 beads
D-: Negative on D1 beads
FN: False negative
FP: False positive
